# Esthetic Rehabilitation through Crown Lengthening Surgery and Conservative CAD/CAM Veneers: A Multidisciplinary Case Report

**DOI:** 10.1155/2016/5720851

**Published:** 2016-09-07

**Authors:** Leandro Passos, Fernando Peixoto Soares, Mauricio Gallo

**Affiliations:** ^1^Department of Prosthetic Dentistry, Federal Fluminense University, Health Institute of Nova Friburgo, School of Dentistry, Rua Doutor Silvio Henrique Braune 22, 28625-650 Nova Friburgo, RJ, Brazil; ^2^Private Practice, Rua Haddock Lobo 1307 Conjunto 101, 01414-003 São Paulo, SP, Brazil; ^3^Private Practice, Rua Germano Wendhausen 203 Sala 301, 88015-460 Florianópolis, SC, Brazil

## Abstract

This case report describes a successful multidisciplinary approach used to improve the smile esthetics of a patient presenting with excessive gingival display, asymmetric gingival margins, and small upper anterior teeth and lower anterior teeth. The treatment combined esthetic crown lengthening, dental bleaching, and restorative dentistry using CAD/CAM veneer. The 6-month follow-up examination confirmed the stability of the modification and absence of adverse effects.

## 1. Introduction

Nowadays, esthetic treatment protocols can resolve any discrepancy and guide decisions in a multidisciplinary approach to obtain satisfactory and predictable results [1]. Accordingly, a digital smile design (DSD) is a useful diagnostic tool to evaluate the tooth size, shape, and position as well as gingival contour [2], enabling us to achieve the expected results for the different aspects [3] of prosthetic planning. In cases of short clinical crowns, an interaction with a periodontist may be necessary to correct abnormalities in the symmetry and contour of the natural or prosthetic teeth by surgical procedures [4].

The emerging concept of no preparation or minimal preparation [5] led to the development of adequate enamel bonding procedures. The color and integrity of dental tissue substrates to which veneer is bound are important for clinical success [6]. The use of 0.3–0.5 mm thick veneer preserves 95%–100% of the enamel volume after the preparation, without exposing the dentin [7]. A number of clinical studies demonstrated that bonded laminate veneer restorations delivered good results over a period of 10 years [8]. Porcelain laminate veneer fabricated with conventional techniques requires an impression of the prepared teeth, an impression of the opposing arch, preparation of casts, and extensive laboratory time [9]. The advances in dental materials and computer technology have made CAD/CAM-fabricated restorations available for dentistry. Furthermore, this approach supports an intraoperative (chairside) workflow for the restoration, fabrication, and insertion of dental implants in a single visit using prefabricated ceramic blocks [10]. The use of CAD/CAM technology to design a restoration in the dental office is more efficient and may be more predictable [11]. In addition, the resistance to fatigue and the tensile strength of CAD/CAM materials were shown to be excellent [12].

The present case report describes a multidisciplinary treatment approach consisting of periodontal crown lengthening with osteotomy, dental bleaching, and CAD/CAM conservative ceramic veneer for the optimal correction of smile esthetics in restorative dentistry.

## 2. Case Presentation

### 2.1. Diagnosis and Treatment Planning

**Figure 1:**
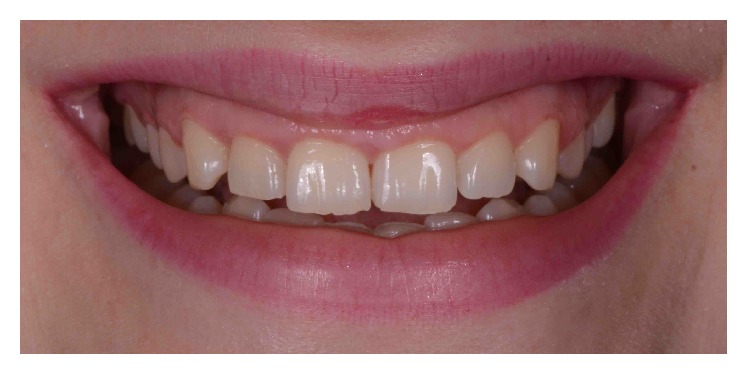
Initial smile.

A 29-year-old systemically healthy woman complained of “a gummy smile” and disliked the shape and size of her superior teeth. Her medical history was unremarkable, and she had no history of smoking or alcohol consumption. Extraoral examination revealed no significant findings. Her face was symmetrical with a straight profile. Her smile line extended to the first molars, and dynamic smiling uncovered approximately 3–5 mm of gingival tissue. However, the patient exhibited a slight asymmetry of the gingival margins on the upper right and left first premolars, with excessive overall gingival display (Figure 1).

**Figure 2:**
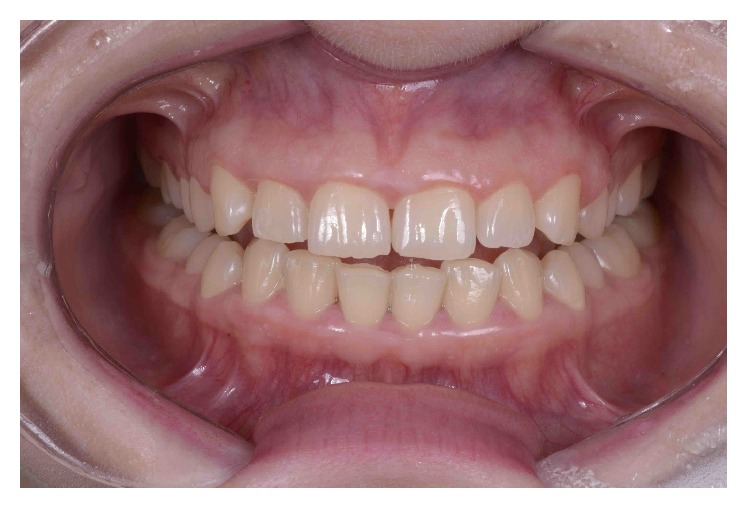
Initial intraoral panoramic view.

The patient underwent a comprehensive clinical examination (Figure 2), as previously described [13]. An assessment of the occlusion and masticatory system was conducted to determine the health of the temporomandibular joints (TMJs), muscles of mastication, and occlusal function. Smile esthetics were analyzed in terms of the initial shade, dental/facial midline, width-to-height ratio of the anterior teeth, buccal corridor, curve of Spee relative to the lower lip in a smile and the free gingival margins, and incisal edge position relative to the lips in repose or full smile and to the F/V sounds. Complete periodontal examination included probing depth, clinical attachment level, bleeding on probing, plaque index, and crown and bone height. The examination was completed with preliminary photographs, X-rays and CT-scan analyses, and diagnostic casts.

**Figure 3:**
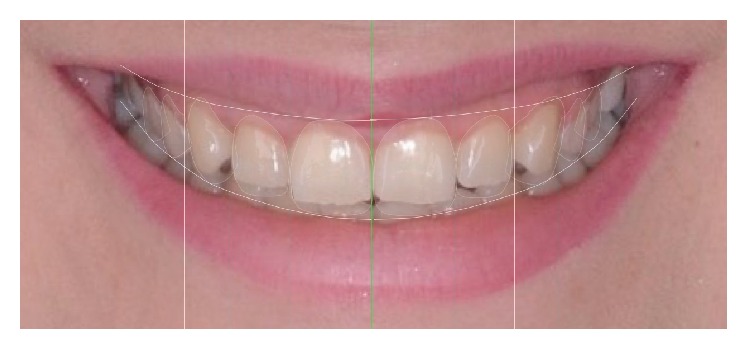
Design of new teeth and gingival combined contour in DSD-based planning phase.

The comprehensive examination indicated that TMJs and muscles of mastication were normal. Occlusal findings showed Class I occlusion with anterior protrusive guidance and canine-protected guidance without crossover contact of the laterals in excursive bilaterally. The dental midline was in alignment with the facial midline. However, the incisal edge of the maxillary central incisors and the occlusal plane were not esthetically acceptable for the upper lip during repose and the lower lip at full smile. The width and length of the central incisor were 8.9 mm and 8.3 mm, respectively, with a ratio of 107% (Figure 3). The displayed free gingival margins of the maxillary central incisors were not in an esthetically acceptable position relative to the upper lip at full smile. The resulting excessive gingival display and slight uneven gingival margins were among the main complaints of the patient. The initial shade was Vita A3.

**Figure 4:**
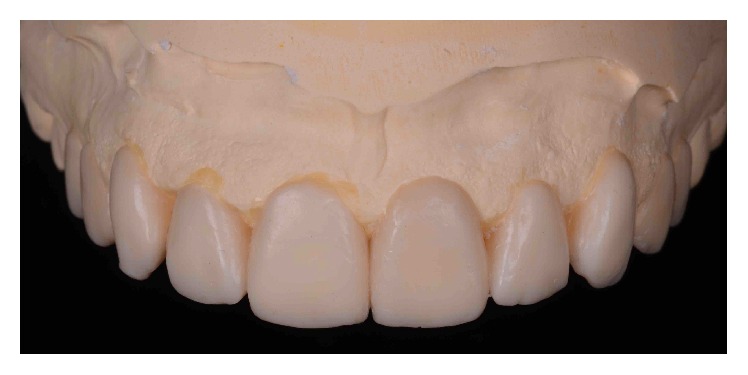
DSD-based diagnostic wax-up.

This information was used to generate a DSD-based diagnostic wax-up mounted on a semiadjustable articulator to visualize the “ideal” tooth shape, potential smile frame, and gingival contour, thereby producing a clear image of the potential restorative outcome to the prosthodontist and periodontist (Figure 4).

After the diagnosis and case analysis, the treatment plan discussed with the patient was as follows: surgical crown lengthening with flap surgery and bone recontouring on teeth 34–44 as well as teeth 15–25 based on the diagnostic wax-up and home bleaching using trays, direct composite restoration of teeth 31 and 41, and indirect restoration of teeth 15–25 with conservative preparation for CAD/CAM feldspathic veneers.

### 2.2. Surgical Technique

**Figure 5:**
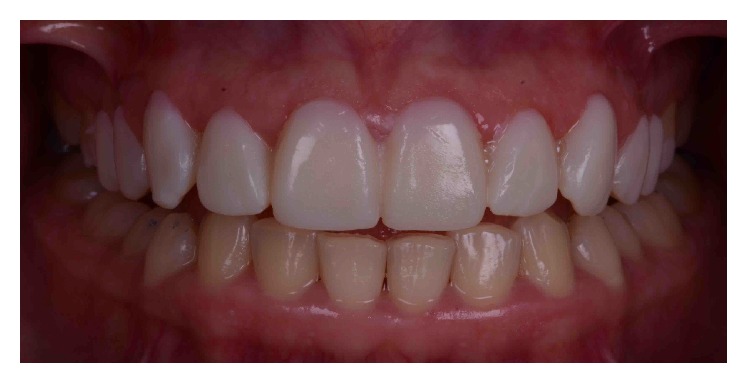
Intraoral try-in of an acrylic template based on the DSD diagnostic wax-up.

**Figure 6:**
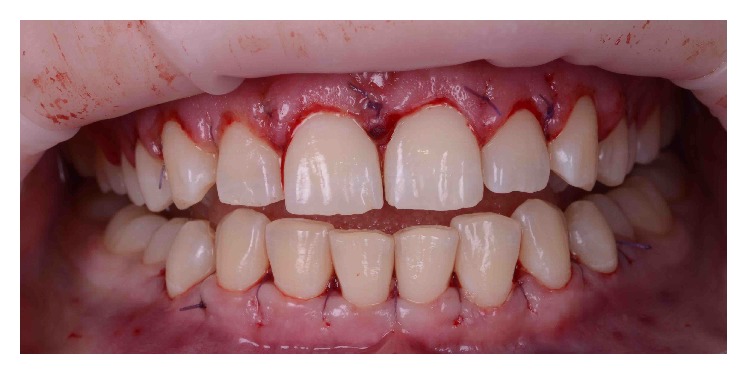
Completed crown lengthening surgery and suture.

The DSD diagnostic wax-up (Figure 5) was used to generate the surgical template that guided the initial incisions [14] and to determine the dimensions of the new clinical crown from the upper right second premolar to the upper left second premolar. The surgical procedures were conducted from the lower first right premolar to the lower first left premolar. First, internal bevel incisions were made according to the surgical guide, and then the collar tissue was removed. The full-thickness flap was elevated to expose the bone crest levels and cementoenamel junctions. Bone remodeling was performed with surgical mini chisels to restore the biologic width. Finally, the flaps were repositioned and sutured (Figure 6). After the surgery, the patient received anti-inflammatory medication (20 mg piroxicam per day for 3 days) and antibiotics (1500 mg amoxicillin per day for 7 days). The sutures were removed 1 week after the surgical procedure. The patient was instructed how to maintain a rigorous regimen of plaque control throughout the treatment period and the 6-month follow-up period.

### 2.3. Bleaching Technique

Once complete healing was confirmed (30 days), the patient conducted dental bleaching at home (10% carbamide peroxide) for a period of 4 weeks. This treatment produced a shade of Vita A1, which was acceptable according to the patient.

### 2.4. Teeth Preparation

Nine weeks after crown lengthening, less invasive procedures optimizing enamel preservation were performed from the upper right second premolar to the upper left second premolar, as previously described [15]. First, we conducted the intraoral fabrication and bonding of an acrylic template using the DSD diagnostic wax-up as a template [16]. The remodeled teeth segments were prepared using round calibrated diamonds guided by the acrylic template. The interproximal contacts were preserved in all preparations. Second, a small diameter dental retraction cord #000 (Ultrapak, Ultradent Inc., South Jordan, Utah, USA) was placed at the bottom of the sulcus to obtain adequate gingival displacement. The cord was left in the sulcus during the entire surface finishing and scanning procedures to provide correct moisture control.

**Figure 7:**
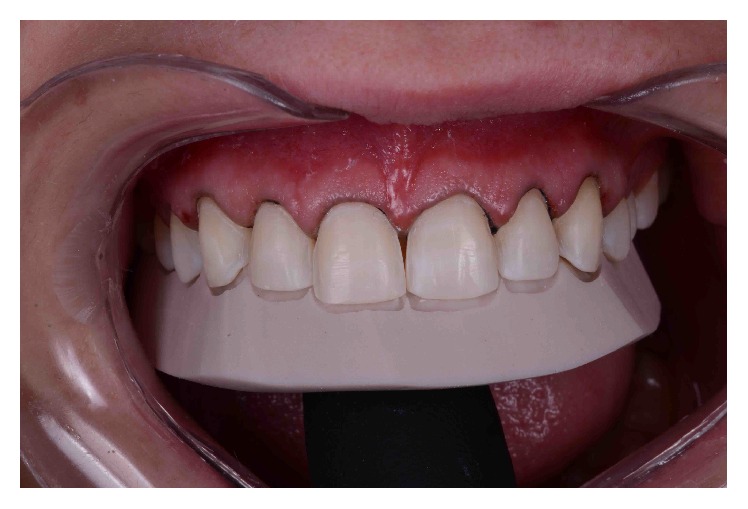
Retracted gingiva and finished teeth preparation.

**Figure 8:**
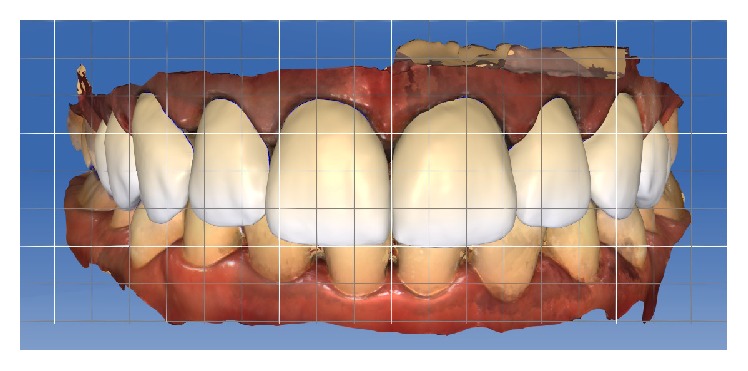
Design of the restorations in CAD/CAM software.

Surfaces of all preparations were finished using stone burs of micrograined aluminum oxide grit (DH Stone PW1114PA, Dhpro Rhadartrade Comercial Importadora de Peças LTDA, Paranaguá, Paraná, Brazil) and polishing disks (Sof-Lex Extra-Thin XT disks #2382SF and #2382F, 3M ESPE Dental, St. Paul, MN, USA) to optimize smooth surfaces for scanning and adhesive cementation (Figure 8). Finally, ultrasonic finishing was performed using specific tips (CR1, CR4, and CR12F. CVDentus, Clorovale Diamentes S/A, São José dos Campos, São Paulo, Brazil), which are considered an alternative approach for minimally invasive tooth preparation procedures [17]. Before the scanning procedures, a retraction paste (3M ESPE Astringent Retraction Paste, 3M ESPE Dental, St. Paul, MN, USA) was applied on all prepared teeth, and the paste was left in place for 2 min. Water spray and high-volume evacuation were used to remove the paste from the sulcus. The gingiva was adequately retracted, the sulcus was dry, and the margins of the preparations were clearly visible (Figure 7).

### 2.5. CAD/CAM Procedures and Restoration Design

The CAD/CAM software (CEREC 4.4, Sirona Dental Systems, Bensheim, Germany) only allows veneer restorations up to the first premolars. Therefore, the CAD/CAM software in biogeneric copy mode was used to perform veneers restorations of teeth 14–24, whereas inlay restorations were selected for teeth 15 and 25. Before teeth preparation, video images of the intraoral acrylic template that reproduced the diagnostic DSD wax-up were acquired using a CAD/CAM tip (Omnicam, Sirona Dental Systems, Bensheim, Germany) for the biogeneric copy veneer restorations and inlay restorations.

The data acquired from the prepared teeth indicated a close correlation between the two models, as the restoration design exhibited the same dimensions as the mock-up scanned previously. The steps performed by the software to build the virtual crown are described below. The model axis was determined by positioning the models according to the midline, inclination, and alignment of the anterior teeth. Then, the software adequately interpreted the images in the initial proposal. The margins were homogeneously delimited, the insertion axis was determined, and the possible retention areas were avoided. Moreover, the Biogeneric Copy Line was determined to enable the design of the Biogeneric Copy veneers.

**Figure 9:**
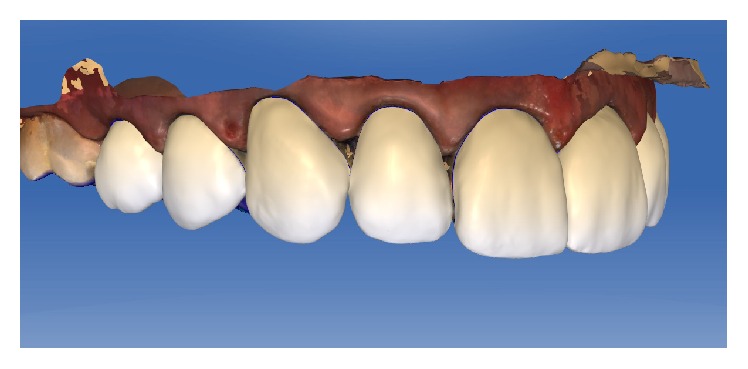
Design of the restorations in CAD/CAM software (lateral view).

**Figure 10:**
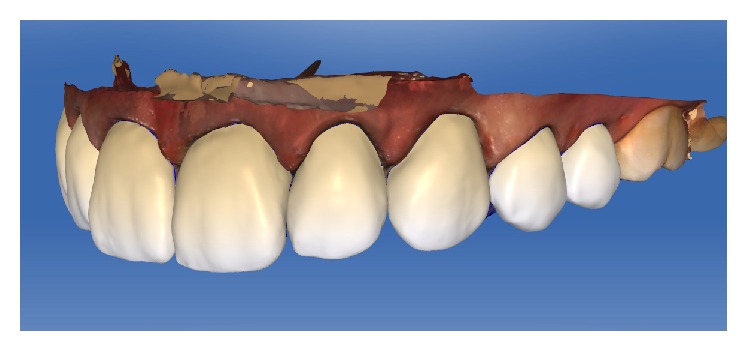
Design of the restorations in CAD/CAM software (lateral view).

**Figure 11:**
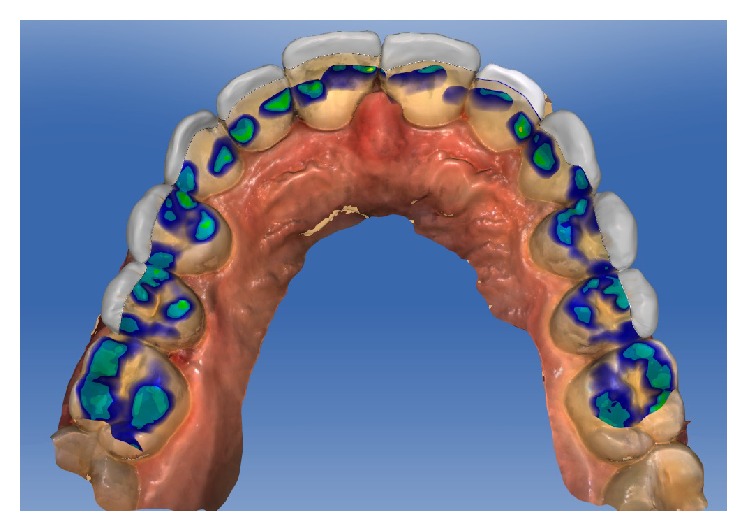
Design of the restorations and occlusal contacts in CAD/CAM software.

The parameters of the veneer-type restorations were as follows: spacer, 60 *μ*m; veneer thickness, 350 *μ*m; occlusal-milling offset, 0 *μ*m; margin thickness, 50 *μ*m; Consider Instrument Geometry, Yes; and Remove Undercuts, Yes. The parameters of the inlay-type restorations were as follows: spacer, 60 *μ*m; marginal gap of adhesive cement, 30 *μ*m; occlusal-milling offset, 0 *μ*m; proximal contact strength, 0 *μ*m; occlusal contact strength, 0 *μ*m; dynamic contact strength, 0 *μ*m; minimum radial thickness, 350 *μ*m; minimum occlusal thickness, 350 *μ*m; marginal thickness, 50 *μ*m; Consider Instrument Geometry, Yes; and Remove Undercuts, Yes. No design modification was made in any of the samples (Figures 8, 9, 10, and 11).

### 2.6. Crown Fabrication

Ten monolithic veneers were fabricated from feldspathic ceramic (10 Vitablocs TriLuxe Forte, Shade A1 on Vitapan, 14/14 mm, Vita Zahnfabrik, Bad Sackingen, Germany). Restorations were milled using a milling unit (CEREC MC XL, Sirona Dental Systems, Bensheim, Germany) in the one-step mode, using a Step Bur 12 (Sirona Dental Systems, Bensheim, Germany) and a Cylindrical Pointed Bur 12S (Sirona Dental Systems, Bensheim, Germany). The cutting diamonds were replaced after milling eight veneers.

### 2.7. Crown Finishing

A certified dental technician (CDT) conducted delicate manual surface enhancement of each restoration through staining, glazing, and polishing steps to improve the texture, color, value, chroma, and gloss. One glaze cycle was done using manufacturer's glazing kits according to manufacturer's instructions.

### 2.8. Cementation

Before cementation, marginal adaptation was verified with a probe using dental loupes (Eyemag Smart Medical Loupes 2.5x magnification, Carl Zeiss Meditec AG, Jena, Germany). For the try-in, the teeth were cleaned with pumice and dried, and then a transparent try-in paste was applied on the intaglio surface of each veneer (Variolink Veneer Shade HV+1 try-in paste, Ivoclar Vivadent, Liechtenstein).

Once the patient approved the restorations, the restorations and teeth were prepared for bonding, according to manufacturer's instructions. The internal surfaces of the feldspathic ceramic restorations were treated with 5% hydrofluoric acid etching gel (Power C Etching 5%, BM4, Brasil Materiais e Instrumentais LTDA, Palhoça, Santa Catarina, Brazil) for 60 s and cleaned using a water spray, followed by ultrasonic cleaning (L100, Schuster Equipamentos Odontológicos, Santa Maria, Rio Grande do Sul, Brazil) in distilled water for 60 s [18]. After the restorations were dried for 20 s, a silane coupling agent (Monobond S, Ivoclar Vivadent, Liechtenstein) was applied to the internal surfaces of all veneers and air-dried for 5 s. Then, a coat of adhesive (Adhese Universal Vivapen, Ivoclar Vivadent, Liechtenstein) was applied to the inner surface of the restorations and left uncured.

The enamel surfaces of the teeth were etched with 37% phosphoric acid for 30 s, washed for 60 s, and gently dried. Then, a universal dental adhesive (Adhese Universal Vivapen, Ivoclar Vivadent, Liechtenstein) was applied and left uncured.

**Figure 12:**
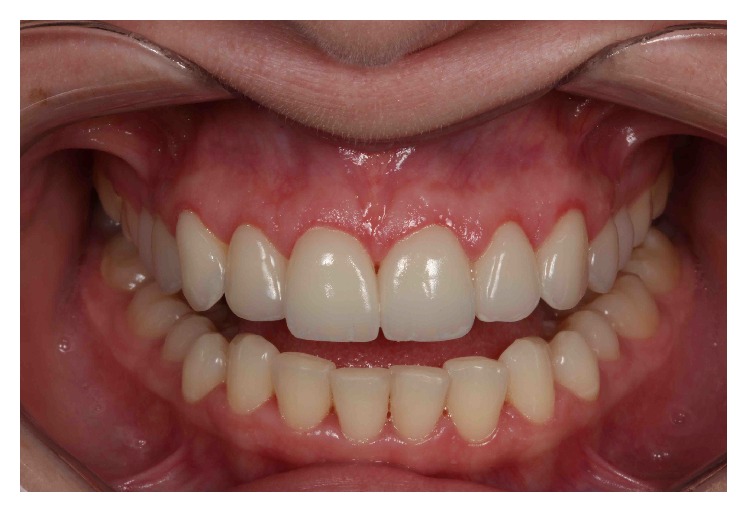
Immediate postoperative intraoral panoramic view.

**Figure 13:**
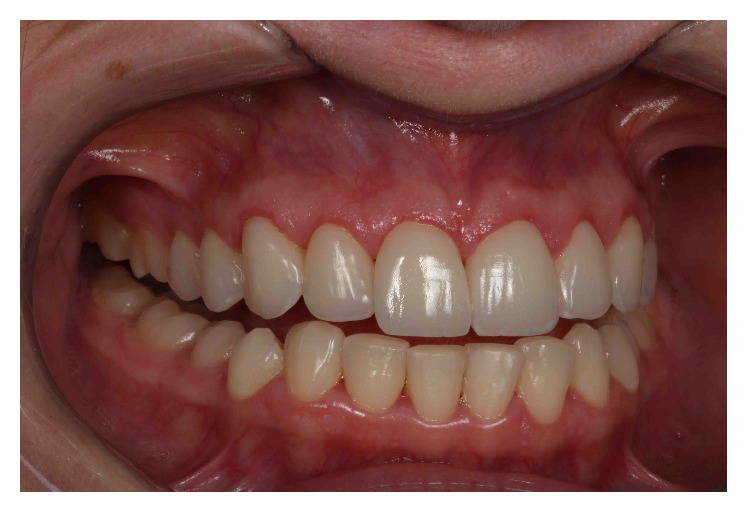
Immediate postoperative intraoral lateral view.

**Figure 14:**
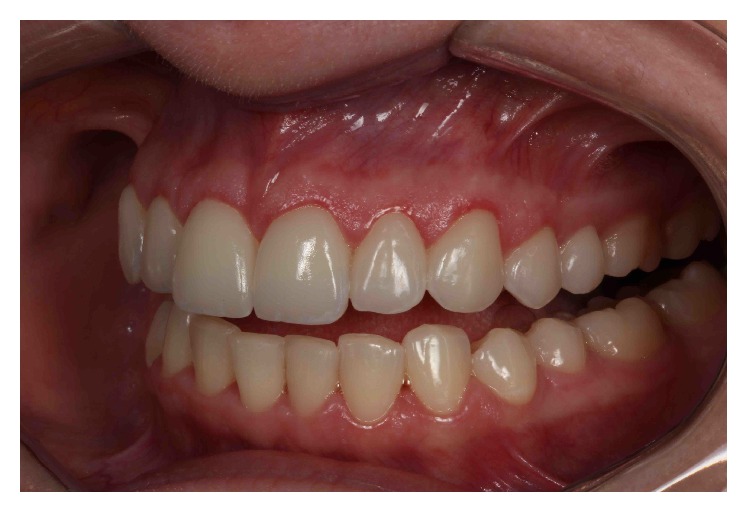
Immediate postoperative intraoral lateral view.

**Figure 15:**
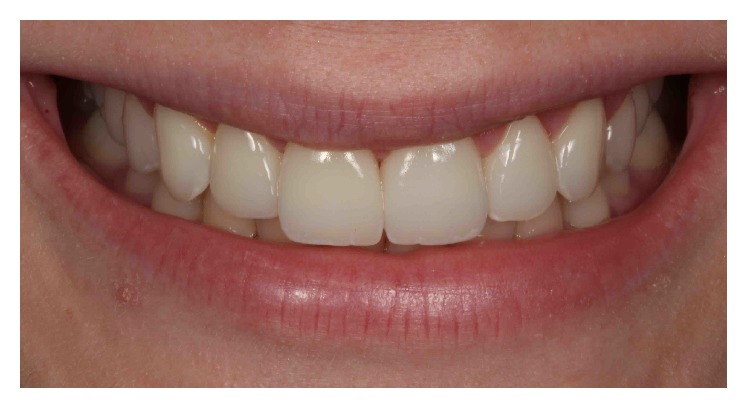
Final smile.

A thin layer of resin cement (Variolink Veneer Shade HV+1, Ivoclar Vivadent, Liechtenstein) was directly applied to the inner surface of the veneers. Then, the restorations were slowly seated on their respective teeth preparations. Pressure was applied to facilitate adaptation under a flow of the luting agent. While holding the veneers in place, excess resin cement was carefully removed using a sickle-shaped scaler (Novatech Cement Remover, Hu-Friedy Co., Chicago, USA). Glycerin gel was applied at the margins to remove the oxygen inhibition layer at the interface. Then, LED light curing was performed on the facial, incisal, and palatal sides for 20 s on each side (Valo, Ultradent Products Inc., South Jordan, Utah, USA) at 1,000 mW/cm^2^. The entire cementation procedure required multiple steps, starting with the second premolars and repeated until central incisors. Following photopolymerization, the remaining cement was removed with a surgical blade #12 and dental probe. Flossing was performed in the interproximal areas to confirm patency at the contact points. The margins were finished and polished as needed with diamond burs, rubber points, and diamond polishing paste (Figures 12, 13, 14, and 15).

**Figure 16:**
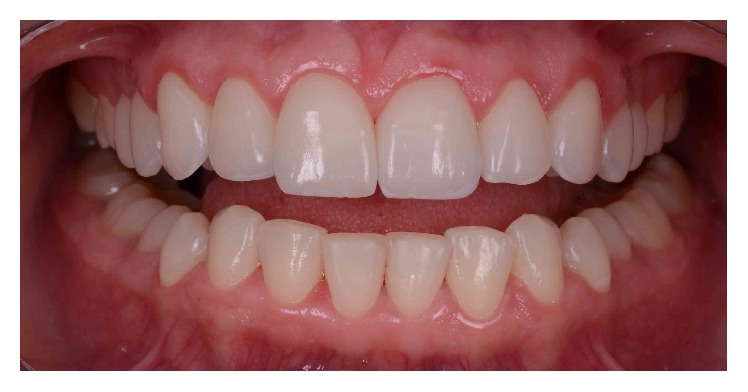
Intraoral panoramic view, 6 months after the periodontal and restorative treatment.

**Figure 17:**
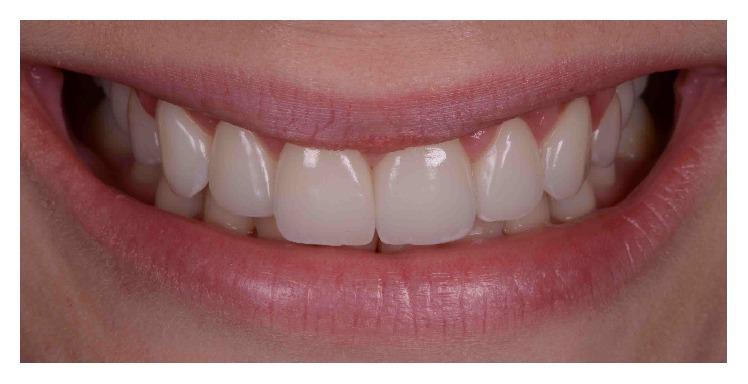
Smile, 6 months after the periodontal and restorative treatment.

The 6-month follow-up examination confirmed the stability of the crown lengthening surgery and restorative results (Figures 16 and 17).

## 3. Discussion

DSD is merely a useful diagnostic tool to plan dentogingival alterations; after the planning, the fabrication of an acrylic template based on a DSD diagnostic wax-up provides the final preview for approval. Then, some artistic modifications should be considered for each patient to harmonize the dental composition to the facial structure [19].

Osseous resection is recommended to obtain a stable improvement of the smile [20] when crown lengthening invades the 3 mm mean dimension of the biologic width. Tomographic examination supports the identification of bone crest location in relation to the cementoenamel junction, allowing a precise surgical resection to optimize esthetics [13]. In this present clinical case, we elected to raise a full-thickness flap and change the bone contour in all superior and inferior areas to restore the biologic width and assure a stable result over time.

A single visit protocol for the fabrication of a porcelain veneer allows better control of the shade and contour and is less time-consuming for the patient and clinician. Single or multiple anterior porcelain laminate veneer restorations can be fabricated with this technique [21]. However in the present case, the planning, surgical pre- and postprocedures, and bleaching treatment required more than one visit. It is also important to note that although saving time and cost is compelling, the technique does require a technical appreciation for the contouring and color matching of the restorations. If the clinician does not have the time or skills to generate highly esthetic restorations, hiring a CDT should be considered.

The Vitablocs TriLuxe Forte are composed of multishade layers and provide a gradient of colors and translucencies. In addition, they exhibit good mechanical properties, with a reported flexural strength of 100–160 MPa when they are glazed [22]. This feldspathic ceramic material possesses excellent esthetic properties, and they are recommended to fabricate veneers, inlays/onlays [23], and single anterior/posterior crowns [24]. When they are used in the premolar region, their fracture load is similar to that of natural teeth [18]. Given these characteristics [25] and excellent optical integration [26], we selected this material to fabricate veneers instead of using lithium-disilicate blocks that, despite high resistance to flexure, are monochromatic and present low fluorescence [26].

The different shades of resin cements may affect the final color of the ceramic veneers. Therefore, it is very important to select the matching color of try-in paste before the cementation [27]. Furthermore, a study showed that the color of CAD/CAM porcelain veneer is significantly affected by its thickness at 0.3 mm, but not in the range of 0.5–0.7 mm [28]. In the present case, the minimum veneer thickness allowed by the CAD/CAM software was 0.35 mm, which highlights the importance of the color and integrity of the dental tissue substrates to which veneers are bonded to ensure clinical success [6].

The limitations imposed by the CAD/CAM software do not allow veneer-type restorations beyond the first premolars. Therefore, we selected an inlay-type of the restoration for the second molars, and the software designed a restoration with the veneer-desired shape.

CEREC restorations have an acceptable marginal adaptation and clinical longevity, along with reduced chair time and improved esthetics. The CAD/CAM technologies give dentists the ability to fabricate a chair-side restoration while controlling all essential elements of a restoration, from the contours and occlusion to the finishing and choice of placement, within timeframes comparable to those of conventional methods [29].

## 4. Conclusion

A comprehensive interdisciplinary approach based on an accurate diagnostic allowed the improvement of smile esthetics through a combination of periodontal plastic surgery, dental bleaching, and conservative CAD/CAM laminate veneers.
